# Comprehensive Molecular Characterization of *Escherichia coli* Isolates from Urine Samples of Hospitalized Patients in Rio de Janeiro, Brazil

**DOI:** 10.3389/fmicb.2018.00243

**Published:** 2018-02-16

**Authors:** Ana Carolina C. Campos, Nathália L. Andrade, Mithila Ferdous, Monika A. Chlebowicz, Carla C. Santos, Julio C. D. Correal, Jerome R. Lo Ten Foe, Ana Cláudia P. Rosa, Paulo V. Damasco, Alex W. Friedrich, John W. A. Rossen

**Affiliations:** ^1^Departamento de Microbiologia, Imunologia e Parasitologia, Faculdade de Ciências Médicas, Universidade do Estado do Rio de Janeiro, Rio de Janeiro, Brazil; ^2^Department of Medical Microbiology, University of Groningen, University Medical Center Groningen, Groningen, Netherlands; ^3^Departamento de Controle de Infecções, Hospital Rio Laranjeiras, Rio de Janeiro, Brazil; ^4^Departamento de Doenças Infecciosas e Parasitárias, Universidade Federal do Estado do Rio de Janeiro, Rio de Janeiro, Brazil; ^5^Departamento de Doenças Infecciosas e Parasitárias, Universidade do Estado do Rio de Janeiro, Rio de Janeiro, Brazil

**Keywords:** *Escherichia coli*, urinary tract infections, Brazil, ST131, antibiotic resistance, virulence genes, whole genome sequencing, diagnostic stewardship

## Abstract

Urinary tract infections (UTIs) are often caused by *Escherichia coli*. Their increasing resistance to broad-spectrum antibiotics challenges the treatment of UTIs. Whereas, *E. coli* ST131 is often multidrug resistant (MDR), ST69 remains susceptible to antibiotics such as cephalosporins. Both STs are commonly linked to community and nosocomial infections. *E. coli* phylogenetic groups B2 and D are associated with virulence and resistance profiles making them more pathogenic. Little is known about the population structure of *E. coli* isolates obtained from urine samples of hospitalized patients in Brazil. Therefore, we characterized *E. coli* isolated from urine samples of patients hospitalized at the university and three private hospitals in Rio de Janeiro, using whole genome sequencing. A high prevalence of *E. coli* ST131 and ST69 was found, but other lineages, namely ST73, ST648, ST405, and ST10 were also detected. Interestingly, isolates could be divided into two groups based on their antibiotic susceptibility. Isolates belonging to ST131, ST648, and ST405 showed a high resistance rate to all antibiotic classes tested, whereas isolates belonging to ST10, ST73, ST69 were in general susceptible to the antibiotics tested. Additionally, most ST69 isolates, normally resistant to aminoglycosides, were susceptible to this antibiotic in our population. The majority of ST131 isolates were ESBL-producing and belonged to serotype O25:H4 and the H30-R subclone. Previous studies showed that this subclone is often associated with more complicated UTIs, most likely due to their high resistance rate to different antibiotic classes. Sequenced isolates could be classified into five phylogenetic groups of which B2, D, and F showed higher resistance rates than groups A and B1. No significant difference for the predicted virulence genes scores was found for isolates belonging to ST131, ST648, ST405, and ST69. In contrast, the phylogenetic groups B2, D and F showed a higher predictive virulence score compared to phylogenetic groups A and B1. In conclusion, despite the diversity of *E. coli* isolates causing UTIs, clonal groups O25:H4-B2-ST131 H30-R, O1:H6-B2-ST648, and O102:H6-D-ST405 were the most prevalent. The emergence of highly virulent and MDR *E. coli* in Brazil is of high concern and requires more attention from the health authorities.

## Introduction

Urinary Tract Infections (UTIs) are one of the most important causes of community and healthcare-associated infections in many clinical onsets worldwide, including Brazil (Terpstra and Geerlings, 2016; Wurpel et al., 2016). Indeed 30–50% of healthcare-associated infections are due to UTIs. This high prevalence is linked to several risk factors, such as catheterization, surgical manipulation and disruption of the urinary tract, diabetes, immunosuppressant drug use, previous admissions, and other comorbidities (Saltoglu et al., 2015; Redder et al., 2016). The risk factors and antibiotic resistance profiles are different for infections acquired in the community or in the hospital environments (Saltoglu et al., 2015). Although in general the majority of UTI cases are uncomplicated, UTIs in hospitalized patients increase the risk for developing sepsis and lead to higher mortality rates (Melzer and Welch, 2013).

*Escherichia coli* is the main etiological agent responsible for 70–90% of all UTIs (Gurevich et al., 2016; Terpstra and Geerlings, 2016). The treatment of patients with UTIs has become increasingly difficult because of the rapid spread of antibiotic resistance (Can et al., 2015). Especially, extended spectrum beta-lactamase (ESBL)-producing *E. coli* are a problem, but an observed rise in fluoroquinolones and aminoglycosides resistance has also significantly contributed to problematic and reduced treatment options for infected patients (Tsukamoto et al., 2013; Bonelli et al., 2014). Several studies have already described the high prevalence of UTIs caused by ESBL-producing *E. coli* in the community and hospitals (Guzmán-Blanco et al., 2014; Gonçalves et al., 2016).

Recently, high antibiotic resistance rates have been associated with specific *E. coli* lineages, such as the multidrug resistant (MDR) sequence type (ST) 131 (Ben Zakour et al., 2016). Particularly, CTX-M beta-lactamase producing *E. coli* of serotype O25:H4 and ST131 is a successful spreading clone (Giedraitiene et al., 2017) strongly associated with the resistance to aminoglycosides and fluoroquinolones. In contrast, other *E. coli* lineages such as ST69, ST73, and ST95, also frequently found as a causative agent of community and hospital acquired UTIs, seem to persist as non-ESBL-producing isolates (Riley, 2014; Doumith et al., 2015).

Extra-intestinal pathogenic *E. coli* (ExPEC), including uropathogenic *E. coli* (UPEC) most commonly associated with human disease, consist of distinct phylogenetic groups with different sets of virulence genes. Previous studies have shown that most ExPEC isolates causing infections belong to phylogenetic groups B2 and D, while isolates in phylogenetic groups A and B1 were mostly identified as commensal *E. coli* isolates (Katouli, 2010). Moreover, pathogenic ExPEC isolates harbor specific virulence genes which confer their pathogenic potential (Cyoia et al., 2015) and are involved in every step in the pathogenicity of ExPEC. Thus, adhesins are a prerequisite to adherence and successful colonization, toxins are responsible for cell damage to urinary tract epithelial cells, and the iron uptake system allows colonization of the urinary tract thereby helping the bacteria to persist (Alizade et al., 2014).

Despite the diversity of ExPEC causing infections, previous studies have shown the connection between specific *E. coli* lineages and their particular resistance profiles, and severity of the infections (Can et al., 2015; Matsumura et al., 2016; Zhang et al., 2016). Thus, defining the genetic background of the pathogen by the identification of a particular ST, its serotype and the detection of resistance genes, can be useful not only for improving further patient treatment but also to allow an improved risk assessment of bacterial infections in the hospitals. The aim of this study is to comprehensively characterize the population structure of *E. coli* from urine samples collected from patients in four hospitals in Rio de Janeiro, Brazil using whole genome sequencing (WGS).

## Materials and methods

### Bacterial isolates

*E. coli* isolates were collected from urine samples of patients admitted to different wards of the Hospital Universitário Pedro Ernesto (HUPE; a 600-bed university hospital) or to one of three small private hospitals (coded Hospital A, Hospital B and Hospital C; see Data Sheet S1). All four hospitals are located in the city of Rio de Janeiro, Brazil. Patients were included regardless the presence of risk factors or observed UTI symptoms. In this study, 107 isolates were collected between November 2015 and November 2016 from the patients (50.60% were from the private hospitals and 49.40% from the public hospital). Eighty-eight percent of the isolates were from female patients. Bacterial isolates were cultured on cysteine lactose deficient medium agar plates (CLED, BD, Germany) till a cell density higher than 10^5^ colony-forming units was obtained. Bacterial cells were stored at −80°C in a Luria-Bertani Broth (LB, Merck, S.A.) with 20% glycerol.

### Bacterial identification and antibiotic susceptibility testing

All isolates were identified using a matrix-assisted laser desorption/ionization time-of-flight (MALDI-TOF) mass spectrometry (Bruker, Germany). Antibiotic susceptibility was performed using VITEK-2 (bioMérieux, Marcy l'Etoile, France) following EUCAST guidelines (v7.1, 2017) and confirmed by E-test (bioMérieux) assays.

### DNA extraction and whole genome sequencing

Total bacterial DNA was extracted from each isolate using the UltraClean® microbial DNA isolation kit (MO BIO Laboratories, Carlsbad, CA, US) following the manufacturer's protocol. A DNA library was prepared for individual samples using the Nextera XT kit (Illumina, San Diego, CA, US) following the manufacturer's instructions. Whole genome sequencing was performed on the Miseq (Illumina) to generate 250-bp paired-end reads to obtain a coverage of at least 60-fold as previously described (Ferdous et al., 2015).

### Assembly and data analysis

*De novo* assembly was performed using CLC Genomics Workbench v10.0.1 (Qiagen, CLC bio A/S, Aarhus, Denmark) using default settings and an optimal word-size. The assembly quality data for all isolates is available in the supplementary data table (Data Sheet S2). Annotation was performed by uploading the assembled genomes onto the RAST server version 2.0 (Aziz et al., 2008). The ST was identified by uploading the assembled genomes in fasta format to the Center for Genomic Epidemiology (CGE) MLST finder website (version 1.7) (Larsen et al., 2012). Presence of antibiotic resistant genes was determined by uploading assembled genomes in fasta format to ResFinder 2.1 (Zankari et al., 2012), the serotyping by using the SerotypeFinder tool (Joensen et al., 2015), and the *fimH* type by uploading the genomes to FimTyper (version 1.0) (Roer et al., 2017) all present through the CGE website.

### Virulence genes, virotype, phylogenetic typing, and analysis

The virulence genes were identified by blasting them against known virulence reference genes (see Data sheet S3) downloaded from the NCBI or ENA database into the CLC Genomics Workbench v10.0.1 (Qiagen, CLC bio A/S, Aarhus, Denmark). In total, 64 virulence genes were investigated and the predictive virulence score was determined using the number of genes found in each isolate. Predictive virulence genes scores were also used to characterize the isolates as ExPEC or UPEC as described by Johnson et al. (2015). The virotype of the ST131 isolates was defined as described by Dahbi et al. (2014). Phylogenetic groups were defined as described by Clermont et al. (2013). To determine the phylogenetic relationship the isolates were uploaded into SeqSphere v.4.1.9 (Ridom, Munster, Germany) and a gene-by-gene typing approach using a 2764-genes core genome (cg) MLST scheme was used as previously described (Ferdous et al., 2016).

### Statistical analysis

The Mann-Whitney test was used to compare the mean of predictive virulence scores (PVS) between the phylogenetic and ST groups. Analysis was performed using GraphPad Prism v7.03 (GraphPad Software, La Jolla, US).

### Nucleotide sequence accession number

The raw data of all whole genome sequenced isolates were deposited in the European Nucleotide Archive under the project number PRJEB23420. See the supplementary data table (Data Sheet S2) for individual accession numbers.

## Results

### Antibiotic resistance pattern

**Figure 1:**
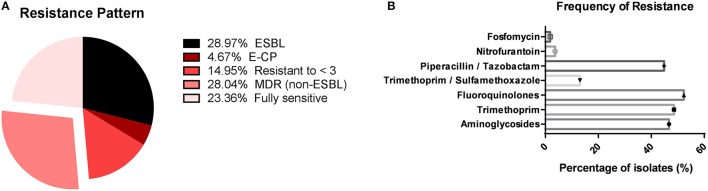
Resistance rates to different classes of antibiotics. **(A)** The percentage of ESBL isolates, *Escherichia coli* carbapenemase producing isolates (E-CP), multidrug resistance isolates excluding ESBL producing ones [MDR (non-ESB)], isolates resistant to less than three antibiotic classes (resistant to < 3) and fully sensitive isolates; **(B)** The frequency for all antibiotic tested, showing the high resistance rate to antibiotics most frequently used in the treatment of UTIs such as aminoglycosides, fluoroquinolones, trimethoprim, and trimethoprim-sulfamethoxazole and a low frequency of resistance to fosfomycin and nitrofurantoin.

MDR was defined as an isolate showing resistance to three or more antibiotic classes. In total, 66 of 107 (61.68%) isolates were MDR and among these isolates, 31 (28.97%) were ESBL-producing, 5 (4.67%) were carbapenemase-producing and 30 (28.04%) were non-ESBL. In addition, 16 (14.95%) isolates were resistant to less than three antibiotic classes and 25 (23.36%) were fully sensitive (Figure 1A). The majority of the isolates was susceptible to fosfomycin (*n* = 105; 98.13%) (Figure 1B). Furthermore, the resistance rate to aminoglycosides (*n* = 50; 46.72%), fluoroquinolones (*n* = 56; 52.33%), trimethoprim (*n* = 52; 48.59%), and trimethoprim-sulfamethoxazole (*n* = 48; 44.85%) was high (Figure 1B), compared to the resistance rates to piperacillin/tazobactam and nitrofurantoin which were 13.08 and 3.73%, respectively. Observed antibiotic resistance profiles including MDR could be linked to the genetic background of *E. coli* isolates (see Data Sheet S1).

### MLST and serotype

**Figure 2:**
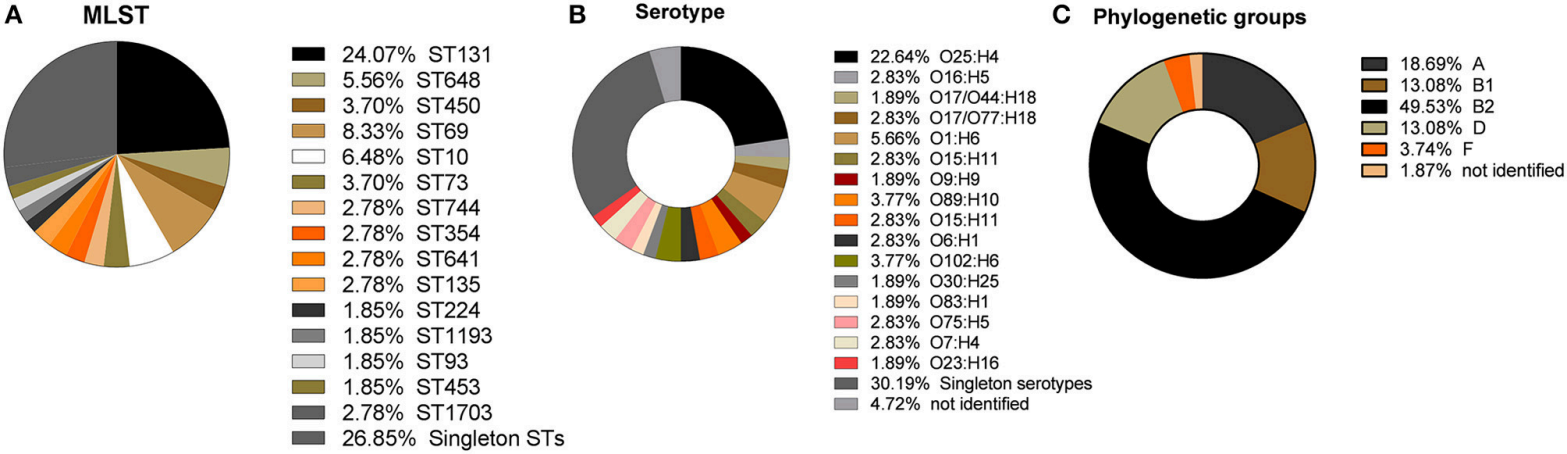
Distribution of sequence types (ST), serotypes, and phylogenetic groups extracted from the whole genome sequence data. **(A)** Percentage of ST lineages found in this study, showing the high prevalence of ST131, ST69, ST10, ST648, ST450, and ST73. Isolates belonging to singleton STs comprise more than one third of the isolates; **(B)** Frequencies of serotypes found showing O25:H4 to be the most frequent serotype; **(C)** Frequencies of the five phylogenetic groups, showing the high prevalence of B2, followed by A, D, and B1 and the low prevalence of isolates belonging to phylogenetic group F.

In this study, 63 (58.87%) isolates were categorized as ExPEC (*n* = 10; 9.34%) or UPEC (*n* = 53; 49.53%) (see Data Sheet S4). Multi locus sequence typing (MLST) was performed and revealed the predominance of six ST groups, namely ST131, ST69, ST648, ST10, ST73, and ST405. ST131 was the most frequent ST found (*n* = 26; 24.07%), followed by ST69 (*n* = 9; 8.33%). In addition, 6 (5.56%) isolates belonged to ST648 and 7 (6.48%) isolates to ST10. ST73 and ST405 were both represented by 4 (3.70%) isolates. Of all the isolates, 29 (26.85%) were singletons representing their own sequence type (Figure 2A). Serotype O25:H4 was the most frequently found (*n* = 24;22.64%) (Figure 2B). Of the ST131 isolates, 92.30% (*n* = 24) belonged to the most frequently found serotype O25:H4 and the other two isolates belonged to serotype O16:H5. All ST405 isolates were serotype O102:H6 and all ST648 isolates were of the O1:H6 serotype. Most isolates of the ST69 group belonged to serotypes O17/O77:H18 (*n* = 4; 44.44%) or O17/O44:H18 (*n* = 2; 22.22%). Other serotypes found in more than 1% of the isolates were O89:H10 (*n* = 4; 3.77%), O102:H6 (*n* = 4; 3.77%), O16:H5 (*n* = 3; 2.83%), O15:H11 (*n* = 3; 2.83%), O6:H1 (*n* = 3; 2.83%), O75:H5 (*n* = 3; 2.83%), O7:H4 (*n* = 3; 2.83%). The other isolates (*n* = 32; 30.19%) had a unique serotype (Figure 2B).

### Phylogenetic analysis

**Figure 3:**
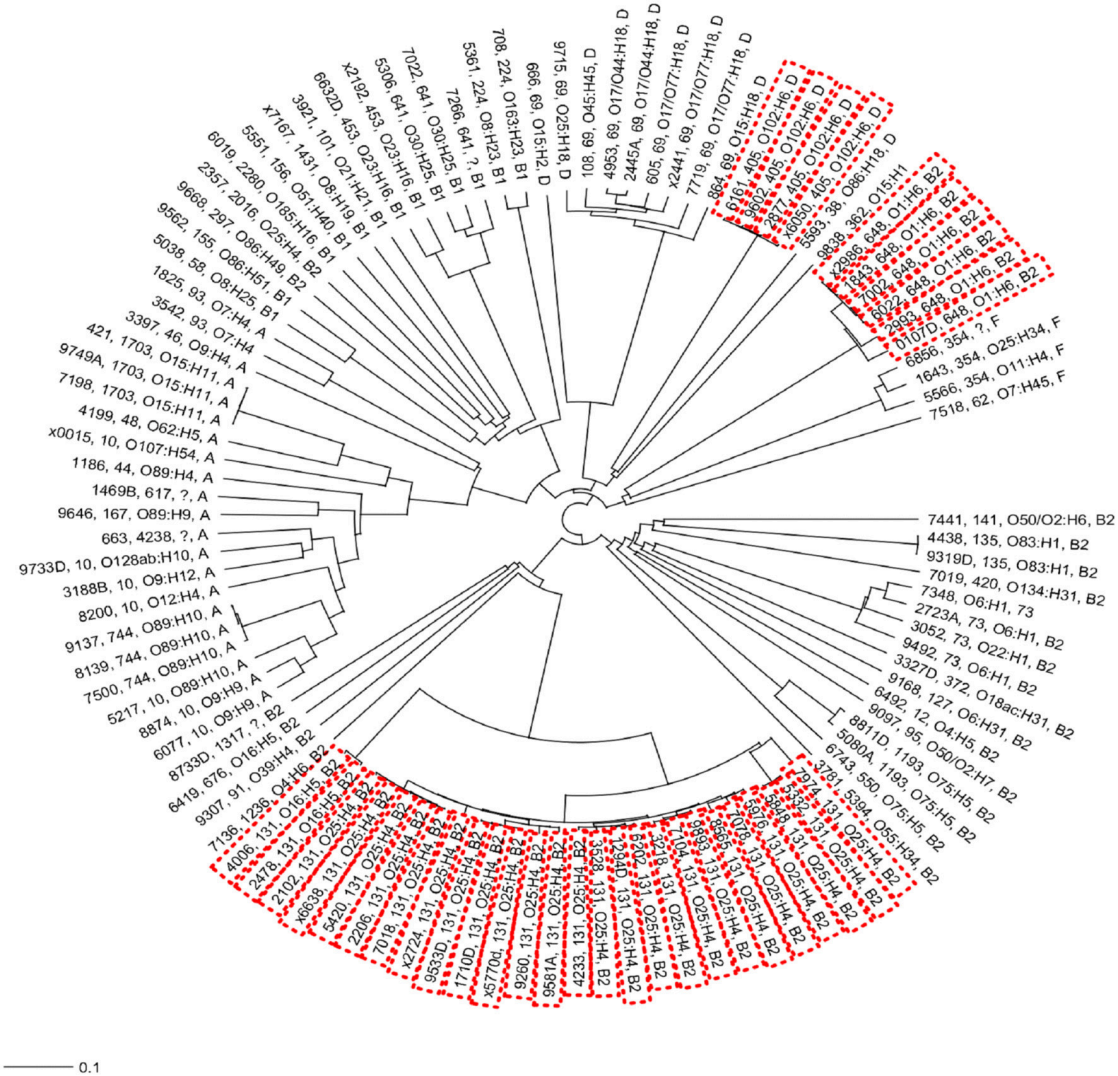
Neighbor-joining (NJ) phylogenetic tree of *Escherichia coli* isolates based on a 2764-genes core genome MLST scheme. High-risk clonal groups are indicated by red doted boxes. For all isolates the phylogenetic groups, serotype and ST group is indicated unless the typing could not be identified from the whole genome sequence data.

In the present study, the most frequently found phylogenetic group was B2 (*n* = 52; 49.53%), followed by phylogenetic groups A (*n* = 20; 18.69%), D (*n* = 14; 13.08%), B1 (*n* = 14; 13.08%), and F (*n* = 4; 3.74%; Figure 2C). For 1.87% of the isolates it was not possible to identify the phylogenetic group (Figure 2C). All ST131, ST73, and ST648 isolates belonged to phylogenetic group B2 while ST69 and ST405 isolates belonged to phylogenetic group D. The isolates of ST10, ST1703, ST744 were classified in the phylogenetic group A and the ST354 isolates were classified in phylogenetic group B1 (see Data Sheet S4). The other isolates represented by a diversity of ST groups were classified into different phylogenetic groups. We investigated the genetic relationships of the sequenced isolated based on their core genome. Not surprisingly, the isolates of the same ST were genetically related and formed ST specific cgMLST clusters (Figure 3). The ST131 isolates with serotype O25:H4 showed less genetic diversity and clustered closely to each other in the cgMLST phylogenetic tree. In general, the ST131 isolates were more closely related with each other while the isolates within ST69 were more diverse. On the other hand, the ST131 isolates could be separated by their serotype and O16:H5/ST131 isolates clustered separately from O25:H4/ST131 ones. Based on the core genome analysis the same was observed for isolates belonging to ST405, ST1703, and ST648 that clustered according to their ST and within such cluster isolates showed a high degree of genetic relatedness. Observed genetic relationships between isolates were independent from their hospital origin.

### Clonal associations of *blaCTX-M*

Whole genome sequencing data was used to screen for the presence of genes responsible for the ESBL phenotype. This analysis revealed that 30 of the 31 (96.77%) ESBL-producing isolates contained a gene encoding a beta-lactamase of the *bla*_CTX−M_ type. In addition, two isolates were AmpC beta-lactamase producing and contained the *bla*_CMY−2_ gene. In the CTX-M positive isolates, *bla*_CTX−M−15_ was the most frequently found variant (*n* = 17; 53.12%) followed by *bla*_CTX−M−8_ (*n* = 5; 15.62%). The majority of *bla*_CTX−M−15_ isolates belonged to O25:H4/ST131, and all the isolates that were CTX-M-15-producing belonged to high risk clonal groups (O25:H4/ST131, O1:H6/ST648, or O102:H6/ST405). Among the singleton isolates 17.24% (*n* = 5) were ESBL-producing, and carried different CTX-M genes (Table 1). Interestingly, the CTX-M-producing isolates were also frequently found to carry genes associated with aminoglycosides and fluoroquinolones resistance. The carbapenemase-producing isolates contained *bla*_KPC−2_ (5 isolates)_.Twelve_ (70.58%) CTX-M-15-producing isolates were also positive for the *bla*_OXA1_ gene (Table 1 and Data Sheet S5).

**Table 1:** Beta-lactamase genes in carbapenemase and ESBL-producing *E. coli* isolates divided by ST groups.

	***bla*** **genes**										
**STs**	***bla_*CTX*−*M*−15_***	***bla_*CTX*−*M*−14_***	***bla_*CTX*−*M*−8_***	***bla_*CTX*−*M*−2_***	***bla_*CTX*−*M*−1_***	***bla_*CMY*−2_***	***bla_*KPC*−2_***	***bla_*OXA*−1_***	***bla_*TEM*−1*A*_***	***bla_*TEM*−1*B*_***	***bla_*TEM*−1*C*_***
**NUMBER OF ISOLATES^a^ (%)**											
ST131	8 (30.76)	0 (0)	0 (0)	*1 (3.84)*	0 (0)	2 (7.69)	4 (15.38)	7 (26.92)	0 (0)	5 (19.23)	0 (0)
ST648	4 (66.66)	0 (0)	0 (0)	0 (0)	0 (0)	0 (0)	0 (0)	4 (66.66)	0 (0)	1 (16.66)	2 (33.33)
ST405	4 (100)	0 (0)	0 (0)	0 (0)	0 (0)	0 (0)	0 (0)	0 (0)	0 (0)	4 (100)	0 (0)
ST69	0 (0)	0 (0)	1 (11.11)	0 (0)	0 (0)	0 (0)	0 (0)	0 (0)	0 (0)	1 (11.11)	0 (0)
ST1703	0 (0)	0 (0)	1 (11.11)	2 (66.66)	0 (0)	0 (0)	0 (0)	0 (0)	0 (0)	2 (66.66)	0 (0)
ST354	0 (0)	1 (33.33)	0 (0)	1 (33.33)	0 (0)	0 (0)	0 (0)	0 (0)	1 (33.33)	1 (33.33)	0 (0)
ST641	0 (0)	0 (0)	0 (0)	0 (0)	1 (33.33)	0 (0)	0 (0)	0 (0)	0 (0)	0 (0)	0 (0)
Singleton STs	0 (0)	1 (33.33)	2 (6.89)	0 (0)	0 (0)	0 (0)	1 (3.44)	0 (0)	1 (33.33)	1 (16.66)	0 (0)

a *Please note that only isolates that have the ESBL phenotype are included in this table*.

### *Escherichia coli* ST131

UPEC strains produce different adhesins and fimbriae, including type 1 fimbriae. The FimH protein is the adhesive subunit of type 1 fimbriae that is used for epidemiological typing of UPEC. In this study, three *fimH* types were identified among the ST131 isolates, two O25:H4/ST131 isolates belonged to *fimH*22, *two* O16:H5/ST131 isolates to *fimH*41 while the majority of O25:H4/ST131 isolates (*n* = 22) belonged to *fimH*30 (Table 2). The virulence genes (*afa/draBC, iroN, sat, ibeA, papGII, papGIII, cnf-1, hlyA, cdtB, neuC-K1, kpsMII-K2, kpsmII-K5*) were used to determine the virotype of ST131 isolates based on the virulence profile. O25:H4/ST131 isolates belonged to different virotypes, i.e., 7 (26.92%) to virotype A, 1 (3.84%) to virotype B, 14 (53.84%) to virotype C, and 4 (15.38%) to virotype D. Isolates belonging to virotype C could be divided into subtypes C2 (*n* = 6) or C3 (*n* = 3), whereas five isolates could not be further subtyped. The only two isolates with serotype O16:H5/ST131 were classified as virotype A (see Data Sheet S6). Almost all O25:H4/ST131 isolates were resistant to fluoroquinolones, whereas the O16:H5/ST131 isolates were susceptible to this antibiotic. The *bla*_CTX−M_ gene was most prevalent in O25:H4/ST131 *fimH30* fluoroquinolone resistant (O25:H4/ST131-H30-R) isolates belonging to virotype C. Within ST131, *bla*_CTX−M15_ was confined to the H30-R sub-clone known as O25:H4/ST131-H30-Rx, represented by 9 (34.61%) isolates (Table 2).

**Table 2:** Distribution of *fimH* types among ST131 *Escherichia coli* isolates.

**Isolates**	**Phylogenetic group**	***FimH* type**	**Serotype**	**Virotype**	**ESBL genes**	**Fluoroquinolone resistant^a^**
5332	B2	*fimH22*	O25:H4	D	*bla_*CMY*−2_*	Pos
7018	B2	*fimH30*	O25:H4	A	*bla_*OXA*−1_*	Pos
7104	B2	*fimH30*	O25:H4	C2	*bla_*KPC*−2_*	Pos
9260	B2	*fimH30*	O25:H4	C	*bla_*CTX*−*M*−15_*	Pos
3218	B2	*fimH30*	O25:H4	C2	*bla_*KPC*−2_*	Pos
9581A	B2	*fimH30*	O25:H4	C	*bla_*CTX*−*M*−15_*	Pos
x5770d	B2	*fimH30*	O25:H4	C	*bla_*CTX*−*M*−15_*	Pos
x6638	B2	*fimH30*	O25:H4	A	*bla_*CTX*−*M*−15_*	Pos
1294D	B2	*fimH30*	O25:H4	B	*bla_*KPC*−2_*	Pos
2102	B2	*fimH30*	O25:H4	A	*bla_*KPC*−2_*	Pos
1710D	B2	*fimH30*	O25:H4	C	*bla_*CTX*−*M*−15_*	Pos
9533D	B2	*fimH30*	O25:H4	C	*bla_*CTX*−*M*−15_*	Pos
3528	B2	*fimH30*	O25:H4	C2	*bla_*CTX*−*M*−15_*	Pos
7078	B2	*fimH30*	O25:H4	C3	*bla_*TEM*−1*B*_*	Neg
9893	B2	*fimH30*	O25:H4	C2	*bla_*KPC*−2_*	Pos
7974	B2	*fimH30*	O25:H4	D	*bla_*CTX*−*M*−2_*	Neg
4233	B2	*fimH30*	O25:H4	D	*bla_*KPC*−2_*	Pos
5420	B2	*fimH30*	O25:H4	A	*bla_*CTX*−*M*−15_*	Pos
2478	B2	*fimH41*	O16:H5	A	*bla_*TEM*−1*B*_*	Neg
4006	B2	*fimH41*	O16:H5	A	*bla_*TEM*−1*B*_*	Neg
5976	B2	*fimH30*	O25:H4	C3	*bla_*TEM*−1*B*_*	Pos
2206	B2	*fimH30*	O25:H4	A	*bla_*CTX*−*M*−15_*	Pos
8565	B2	*fimH30*	O25:H4	C3	*bla_*TEM*−1*B*_*	Pos
x2724	B2	*fimH30*	O25:H4	C2	*bla_*TEM*−1*B*_*	Pos
6202	B2	*fimH30*	O25:H4	C2	*bla_*TEM*−1*B*_*	Pos
5848	B2	*fimH22*	O25:H4	D	*bla_*CMY*−2_*	Neg

a *Neg. indicates susceptible to fluoroquinolones and Pos. indicates resistant to fluoroquinolones*.

### Virulence genes

*E. coli* isolates were screened for the presence of virulence genes potentially associated with UTIs. In total, 64 virulence genes were investigated among the analyzed isolates (Data Sheet S7). Most frequently virulence genes found were those involved in the iron uptake system, such as *fhuE* (ferrichrome receptor) (*n* = 105; 98.13%), *tonB* (TonB protein) (*n* = 105; 98.13%), *fepA* (ferrienterobactin receptor precursor) (*n* = 105; 98.13%), *fhuA* (Ferrichrome receptor precursor) (*n* = 101; 94.39%), and *fyuA* (yersiniabactin receptor) (*n* = 78; 72.89%). Less frequently found genes involved in the uptake of iron were: *iroN* (enterobactin siderophore receptor protein) (*n* = 23; −21.49%), *iha* gene (encoding the adherence protein) (*n* = 34; 31.77%), and *iutA* (aerobactin receptor) (*n* = 52; 48.59%). The presence of the gene cluster *papAH* (P fimbria structural subunits) known to be responsible for P fimbria formation was present in 48 isolates. Interestingly, in 12 isolates *papGII* (a P adhesin variant) was identified and in 15 isolates (14.01%) *papGIII* was found. Other virulence genes, encoding adhesins, detected were: the *fimH* gene in 104 isolates (97.19%) and the *lpfA* gene (encoding for the long polar fimbriae) in 31 isolates (28.97%). The gene encoding a toxin *hlyD* (hemolysin D) was identified in 105 isolates tested (98.13%), however other toxin genes were less frequently found and included *sat* (*n* = 30; 28.03%), *senB* (*n* = 21;19.69%), and *cnf*-1 (*n* = 9; 8.41%). Other virulence genes identified in the majority of isolates were *malX* (pathogenicity island marker) (*n* = 103; 96.26%), *gad* (glutamate decarboxylase) (*n* = 88; 82.24%), *iss* (increase serum survival) (*n* = 82; 76.63%), *ompT* (outer membrane protease) (*n* = 72; 67.28%), *traT* (serum resistance associated) (*n* = 66; 61.68%), and *kpsM* (capsule transport protein) (*n* = 60; 56.07%) (Table 3).

**Table 3:** Prevalence of main virulence genes among *E. coli* isolates in relation to phylogenetic groups and sequence types (ST).

**Virulene factors^a^**	**No. of isolates (%)**										
	**A**	**B1**	**B2**	**D**	**F**	**ST131**	**ST648**	**ST405**	**ST69**	**ST10**	**ST73**
*fhuE*	19 (95)	14 (100)	52 (100)	14 (100)	4 (100)	26 (100)	6 (100)	4 (100)	9 (100)	7 (100)	3 (75)
*tonB*	20 (100)	13 (92.85)	52 (100)	14 (100)	4 (100)	26 (100)	6 (100)	4 (100)	9 (100)	7 (100)	3 (75)
*fepA*	19 (95)	14 (100)	52 (100)	14 (100)	4 (100)	26 (100)	6 (100)	4 (100)	9 (100)	7 (100)	3 (75
*fhuA*	20 (100)	14 (100)	48 (92.30)	14 (100)	4 (100)	26 (100)	5 (83.33)	4 (100)	9 (100)	7 (100)	3 (75)
*fyuA*	6 (30)	5 (35.71)	51 (98.07)	14 (100)	2 (50)	26 (100)	6 (100)	4 (100)	9 (100)	3 (42.85)	3 (75)
*iroN*	4 (20)	5 (35.71)	11 (21.15)	1 (7.14)	2 (50)	3 (11.53)	0 (0)	0 (0)	0 (0)	2 (28.57)	3 (75)
*iutA*	6 (30)	2 (14.28)	34 (65.38)	10 (71.42)	2 (50)	23 (88.46)	6 (100)	0 (0)	7 (77.77)	3 (42.85)	0 (0)
*papAH*	2 (10)	1 (7.14)	38 (73.07)	8 (57.14)	1 (25)	21 (80.76)	3 (50)	0 (0)	6 (66.66)	2 (28.57)	4 (100)
*papGII*	0 (0)	0 (0)	11 (21.15)	2 (14.28)	0 (0)	5 (19.23)	3 (50)	0 (0)	2 (22.22)	0 (0)	0 (0)
*iha*	4 (20)	0 (0)	25 (48.07)	6 (42.85)	0 (0)	21 (80.76)	0 (0)	0 (0)	5 (55.55)	2 (28.57)	1 (25)
*IpfA*	0 (0)	14 (100)	7 (13.46)	7 (50)	4 (100)	0 (0)	4 (66.66)	0 (0)	5 (55.55)	0 (0)	0 (0)
*hlyD*	20 (100)	12 (85.71)	52 (100)	14 (100)	4 (100)	26 (100)	6 (100)	4 (100)	9 (100)	7 (100)	4 (100)
*malX*	19 (95)	14 (100)	50 (96.15)	14 (100)	4 (100)	23 (88.46)	6 (100)	4 (100)	9 (100)	7 (100)	4 (100)
*ompT*	19 (95)	14 (100)	47 (90.38)	13 (92.85)	4 (100)	23 (88.46)	6 (100)	4 (100)	9 (100)	7 (100)	4 (100)
*kpsM*	2 (10)	0 (0)	42 (80.76)	13 (92.85)	3 (75)	19 (73.07)	6 (100)	4 (100)	6 (66.66)	1 (14.28)	4 (100)
*afa*	1 (5)	0 (0)	7 (13.46)	0 (0)	0 (0)	7 (26.92)	0 (0)	0 (0)	0 (0)	1 (14.28)	0 (0)
*cnf-1*	1 (5)	0 (0)	8 (15.38)	0 (0)	0 (0)	4 (15.38)	0 (0)	0 (0)	0 (0)	0 (0)	1 (25)
*sfaS*	0 (0)	0 (0)	2 (3.84)	0 (0)	0 (0)	0 (0)	0 (0)	0 (0)	0 (0)	0 (0)	0 (0)
*traT*	12 (60)	6 (42.85)	31 (59.61)	14 (100)	2 (50)	19 (73.07)	6 (100)	3 (75)	8 (88.88)	5 (71.42)	0 (0)
*usp*	0 (0)	0 (0)	41 (78.84)	1 (7.14)	3 (75)	25 (96.15)	0 (0)	0 (0)	0 (0)	0 (0)	4 (100)
***P*-values (PVS)^b^**											
ST73 vs. other STs		S *P* < 0.0001		ST10 vs. other STs		S *P* < 0.0001		Group 1vs. Group 2		NS *P* = 0.2190	
ST131vs. ST69		NS *P* = 0.2444		ST405 vs. other STs		S *P* < 0.0001					
ST648 vs. ST69		NS *P* = 0.9993		Singletons STs vs. other STs		NS S *P* = 0.0.439					

a Genes most frequently found and/or associated with UTIs.

b *Comparison of predictive virulence mean scores between different ST groups between phylogenetic group 1 (isolates that belong to phylogenetic group A or B1) and group 2 (isolates that belong to phylogenetic group B2, D, or F). The statistical tests were performed using Mann-Whitney test and were considerate significant if p < 0.05. Abbreviations used: vs, versus; S, significant and NS, not significant*.

### Association of ST and phylogenetic groups with resistance pattern

**Figure 4:**
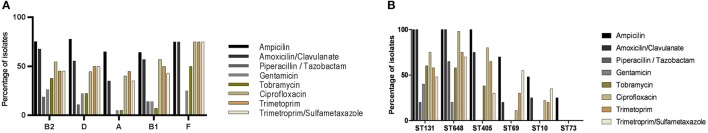
Antibiotic resistance profiles**. (A)** Percentage of isolates resistant to the indicated antibiotics grouped by phylogenetic groups (A, B1, B2, D, or F). **(B)** Percentage of isolates resistant to the indicated antibiotic classes grouped by sequence type (ST). Only the six most prevalent STs are indicated.

The majority of the MDR isolates belonged to ST131, ST648, or ST405 while most non-MDR isolates belonged to ST69, ST10, ST73, or singleton STs. The ST131, ST648, and ST405 isolates also showed a higher resistance rate to other antibiotic classes as ampicillin and amoxicillin/clavulanate (Figure 4A). Among the singleton STs, the number of MDR isolates was low. The phylogenetic groups B2, D, and F were more often found to be resistant to ampicillin, amoxicillin/clavulanate, ciprofloxacin, and trimethoprim than phylogenetic groups A and B1 (Figure 4B).

### Association of ST and phylogenetic group with virulence genes

The main six ST groups identified in this study were compared to evaluate their urovirulence-potential, using the 64 identified virulence genes (Data Sheet S7). Based on the predictive virulence score (PVS) no statistically significant difference was found for ST131 (PVS = 18.3) and ST648 (PVS = 17.6) isolates compared to ST69 (PVS = 17.8) isolates (*p* = 0.2444 and *p* = 0.9993, respectively). In contrast, the ST405 (PVS = 13.0) and ST10 (PVS = 12.7) isolates had lower PVS compared to other STs groups (*p* < 0.0001). The ST73 isolates appeared to have the highest PVS (24.0) compared to other groups (*p* < 0.0001). Interestingly, the PVS for isolates belonging to singleton ST groups scored slightly higher (PVS = 19.0) than isolates belonging to ST131, ST648, ST405, ST69, and ST10 (*p* = 0.0439). When the same analysis was performed on different phylogenetic groups, phylogenetic groups B2, D, and F had higher PVSs than phylogenetic groups A and B1 (*p* = 0.2190), although this was not statistically significant (Table 3).

## Discussion

In this study, a comprehensive molecular characterization of *E. coli* isolated from urine samples of hospitalized patients in hospitals in Rio de Janeiro was performed and showed the presence of successful MDR clones similar to those found in other parts of the world (Riley, 2014). In general, high resistance rates to antibiotics such as cephalosporin, aminoglycosides, fluoroquinolones and trimethoprim often used to treat patients with UTIs were found. The emergence of MDR *E. coli* complicates the treatment of UTIs and is a major concern for hospitals (Flores-Mireles et al., 2015). Our results are in agreement with previous reports from Brazil, showing an increase of resistance rates of *E. coli* to aminoglycosides and fluoroquinolones (Correal et al., 2014; Rodrigues et al., 2016). In addition, the resistance rates to fosfomycin and nitrofurantoin, antibiotics used to treat uncomplicated UTIs, were found to be low in the investigated isolates, consistent with results from previous studies (Michalopoulos et al., 2011; Derakhshandeh et al., 2015).

In our study, 49.53% of the isolates were identified as UPEC and 9.34% were classified as ExPEC (non-UPEC) based on predictive virulence genes score. The other 41.13% could not be typed as ExPEC using this method, indicating that the predictive virulence genes score is not always sufficient for classification of ExPEC as has also been reported before (Berman et al., 2014). In general, ExPEC can be classified into five phylogenetic groups, i.e., A, B (subgroups B1 and B2), D, E, and F, and the majority of the isolates in our study belonged to phylogenetic groups B2 and D. Indeed, other studies, as the ones from Iran and China, show that human pathogenic ExPEC predominantly belong to these two groups (Kazemnia et al., 2014; Tong et al., 2014), that are also considered to be more virulent and more associated with infections than, e.g., phylogenetic groups A and B1 (Lee et al., 2016). In our study, two isolates could not be assigned to any of the phylogenetic groups. This is in agreement with findings of others that assigning isolates to a specific phylogenetic group based on the current guidelines is not always possible (Clermont et al., 2013). The phylogenetic groups B2 and D were more often found to be MDR than the isolates of phylogenetic groups A and B1, which is agreement with other studies (Lee et al., 2016).

In our study population, the two most frequently found *E. coli* lineages were ST131 and ST69, which is in line with previous studies showing the worldwide spread of these STs and their association with UTIs (Peirano et al., 2014; Doumith et al., 2015). ST69 has previously been associated with both community acquired and healthcare associated UTIs (Riley, 2014) and appears to be frequently MDR, due to the presence of a resistance gene cassette (*dfrA17*-*aadA5*) that confers resistance to aminoglycosides and trimethoprim (Riley, 2014). Interestingly, our results showed that ST69 isolates were susceptible to aminoglycosides but had a high resistance rate to trimethoprim. As ST131 has emerged as the most prevalent high-risk lineage among infections caused by *E. coli* (ExPEC), its high prevalence in this study is not surprising. Moreover, the high frequency of the O25:H4/ST131 clonal group was also similar to findings of others in Brazil, Lithuania and the Netherlands (Dias et al., 2009; Overdevest et al., 2015; Giedraitiene et al., 2017). Other ST groups found in this study include ST648, ST405, ST73, and ST10, previously shown to be associated with urinary and blood-stream infections (Peirano et al., 2014; Doumith et al., 2015; Gonçalves et al., 2016; Hertz et al., 2016; Matsumura et al., 2016). Interestingly, in contrast to other studies performed in the UK and Denmark, the high virulent lineage ST73 was found less frequently than ST10, i.e., only in 3.7 and 6.7% of the collected isolates, respectively (Gibreel et al., 2012; Hertz et al., 2016).

ESBL-producing bacterial isolates are of great medical concern in Latin American countries such as Brazil (Bonelli et al., 2014; Sampaio and Gales, 2016). The majority of ESBL-producing isolates in this study carried the *bla*_*CTX*−*M*−15_ gene, different from previous studies, in which *bla*_*CTX*−*M*−2_ and *bla*_*CTX*−*M*−8_ were found most frequently (Bonelli et al., 2014; Guzmán-Blanco et al., 2014). The majority of ESBL-producing isolates in O25:H4/ST131 clonal group were CTX-M-15 producing. The *E. coli* O25:H4/ST131 CTX-M-15 producing isolates were detected in other countries worldwide (Yumuk et al., 2008; Merino et al., 2016) and are known to be associated with increased capacity of plasmid uptake which results in high plasmid diversity despite showing a similar phenotype (Petty et al., 2014). In addition, the O25:H4/ST131 CTX-M-producing isolates in this study were also found to be resistant to gentamicin, tobramycin, and ciprofloxacin. This is similar to data presented in studies worldwide that showed that CTX-M-producing isolates are often MDR (Pitout and Laupland, 2008; Ewers et al., 2014; Ciesielczuk et al., 2015).

In general, higher resistance rates for more than three antibiotic classes were found in isolates belonging to ST131, ST648, and ST405. These results are in agreement with previous studies in the UK and Denmark that showed a broad-spectrum resistance of ST131 *E. coli* (Ferjani et al., 2014; Hertz et al., 2016) and that ST648 and ST405 have mobile elements containing genes that confer resistance to aminoglycosides, sulfonamides, and trimethoprim (Matsumura et al., 2013; Zhang et al., 2016). In addition, the successful spread of the high-risk clone O25:H4/ST131 is largely responsible for the increased prevalence of ESBL-producing isolates. Other examples of *E. coli* high-risk clones include isolates that belong to ST405 and ST648 (Johnson et al., 2013; Mathers et al., 2015). Our results showed that all ST131 isolates belong to phylogenetic group B2 and that all ST405 isolates belong to phylogenetic group D. These groups, often CTX-M-ESBL producing, have been reported as high-risk pandemic clones (Wang et al., 2016; Shaik et al., 2017). Patients carrying such a high-risk isolate that easily spreads can be the cause of outbreaks in hospital settings and should be put into isolation upon admission.

In contrast to findings of others who reported that ST648 isolates belong to phylogenetic group D (Gonçalves et al., 2016; Müller et al., 2016), we found that the ST648 isolates in this study belong to phylogenetic group B2. This classification was based on the observation that in the whole genomes of our ST648 isolates the *yjaA* and *arpA* genes were absent, whereas the *tsp*E4.C2 and *chuA* genes were present. Therefore, they belong to phylogenetic group B2 based on the phylo-typing method described by Clermont et al. (2013). In addition, our ST648 isolates contained a mutation (G → C) in the primer binding site of primer TspE4C2.1b at the position where the most 3' nucleotide of this primer should anneal. This may lead to misclassification of the isolate as belonging to phylogenetic group F instead of B2 when using the PCR-based method for phylo-typing described by Clermont et al. (2013).

The results of this study, show that the majority of O25:H4/ST131 isolates belong to subclone H30-R, whereas part of these isolates belong to subclone H30-Rx (classified as virotype C or A). The rise in fluoroquinolone resistance in the last years is associated with the rapid emergence of this latter subclone that is often MDR (Peirano et al., 2014). It has also been associated with upper UTIs and primary sepsis, and often contains the *aac(6')-Ib-cr* gene (responsible for fluoroquinolone resistance) (Peirano et al., 2014). The evolutionary history of sub-clone H30-Rx is unclear. The most accepted theory to explain the success of its emergence is that it has, as other high-risk bacterial clones, acquired certain adaptive traits and survival skills while acquiring antibiotic resistance and virulence genes located on mobile elements (Woodford et al., 2011; Mathers et al., 2015). Therefore, detailed molecular characterization studies are required to increase the knowledge about the evolution of this subclone (Petty et al., 2014; Matsumura et al., 2016) and to identify specific molecular markers (including resistant/virulence genes and/or specific plasmids) to optimize diagnostics and subsequent antibiotic therapy.

The pathogenicity of UPEC is based on virulence and fitness factors that allow the bacteria to entry, adhere, acquire essential nutrients such as iron, multiply, cause tissue damage, and disseminate in the urinary tract (Subashchandrabose et al., 2014). The most frequently found virulence genes in our isolates were associated with the iron uptake system and adhesins, whereas fimbriae and toxins were less frequently found. These results differ from previous studies where a high frequency of adhesins and toxins genes among UPEC isolates were found (Alizade et al., 2014). Whereas, several studies showed the association between the presence of adhesins and toxins with more complex UTIs (Wiles et al., 2008; Tarchouna et al., 2013), others could not correlate the presence of these virulence genes with the complexity of UTIs (Kudinha et al., 2013; Firoozeh et al., 2014). Most likely, the complexity of a UTI is defined by a combination of virulence genes, including those associated to the iron uptake system and adhesins. Indeed, efficient iron uptake is essential for the bacteria to survive and colonize in a poor iron environment as the urinary tract (Lee et al., 2016). In addition, the presence of adhesins such as *afa, pap, sfa* has been described to be important for invading urinary epithelial cells and in our isolates identified virulence genes *cnf-1* and *hlyA* are essential subsequent dissemination (Lee et al., 2016). Other genes frequently found in our isolates were *ompT, malX, kpsM*, and *traT*. These genes are common virulence genes found in isolates associated with cystitis and pyelonephritis (Firoozeh et al., 2014; Derakhshandeh et al., 2015).

Overall, in our study, virulence genes were most prevalent among B2 isolates, followed by group D and F. In addition, their prevalence among sequence types ST131, ST69, ST1703, ST405, and ST648 was similar. ST73 isolates had a higher PVS compared to the other investigated groups. This is in agreement with findings of others that described *E. coli* ST73 to be a high virulent clone (Alhashash et al., 2016). In addition, ST131-B2 strains have emerged globally causing MDR resistant extraintestinal infections (Johnson et al., 2013). Therefore, MDR isolates belonging to phylogenetic group B2 and clonal group O25:H4/ST131 are considered to form a double threat, because of their high resistance rate and substantial extraintestinal virulence capacity (Ferjani et al., 2014).

In conclusion, a large diversity of *E. coli* isolates causing UTIs was found in urine samples obtained from patients in Rio de Janeiro. The identified STs belonged to the most prevalent clonal groups reported worldwide. Among the investigated isolates the antibiotic resistance rate was high, as was the prevalence of ESBL-producing isolates. This result is associated with the presence of high-risk clones, often MDR, that mainly belong to phylogenetic group B2 D and F, containing a high number of virulence genes. The presence of highly virulent and MDR *E. coli* in Brazilian hospitals is of high concern for health care institutions and requires more attention from the health authorities. Clearly, it has consequences for the treatment of the patients and the outcome of the disease. Therefore, standard implementation of molecular methods to characterize *E. coli* isolates from urine in hospitalized patients is required to optimize diagnostic stewardship, patient treatment and infection control measures.

## Ethics statement

This study was approved by the Pedro Ernesto University Hospital ethical committee according and with Brazilian legislation and receive this register number: CAAE:45780215.8.0000.5259.

## Author contributions

AC: drafting the article, data analysis, and interpratation; NA, CS, and JC: data collection and sample collection; MF and MC: data analysis and interpretation; JL: revision of the article; AR: conception and design of the work; PD: data collection and revision of the article; AF: final approval of the version to be published; JR: critical revision of the article.

### Conflict of interest statement

The authors declare that the research was conducted in the absence of any commercial or financial relationships that could be construed as a potential conflict of interest. The reviewer VG and handling Editor declared their shared affiliation.
