# Corrigendum: Comprehensive Molecular Characterization of *Escherichia coli* Isolates from Urine Samples of Hospitalized Patients in Rio de Janeiro, Brazil

**DOI:** 10.3389/fmicb.2020.599031

**Published:** 2020-10-30

**Authors:** Ana Carolina C. Campos, Nathália L. Andrade, Mithila Ferdous, Monika A. Chlebowicz, Carla C. Santos, Julio C. D. Correal, Jerome R. Lo Ten Foe, Ana Cláudia P. Rosa, Paulo V. Damasco, Alex W. Friedrich, John W. A. Rossen

**Affiliations:** ^1^Departamento de Microbiologia, Imunologia e Parasitologia, Faculdade de Ciências Médicas, Universidade Do Estado Do Rio de Janeiro, Rio de Janeiro, Brazil; ^2^Department of Medical Microbiology, University of Groningen, University Medical Center Groningen, Groningen, Netherlands; ^3^Departamento de Controle de Infecções, Hospital Rio Laranjeiras, Rio de Janeiro, Brazil; ^4^Departamento de Doenças Infecciosas e Parasitárias, Universidade Federal Do Estado Do Rio de Janeiro, Rio de Janeiro, Brazil; ^5^Departamento de Doenças Infecciosas e Parasitárias, Universidade Do Estado Do Rio de Janeiro, Rio de Janeiro, Brazil

**Keywords:** *Escherichia coli*, urinary tract infections, Brazil, ST131, antibiotic resistance, virulence genes, whole genome sequencing, diagnostic stewardship

In the original article, there was a mistake in Table 2 and Supplementary Data Sheet 1, S.6 as published. The virotype of the isolate 4233 is not typeable. The corrected Table 2 and Supplementary Data Sheet 1, S.6 appear below.

**Table 2:** Distribution of *fimH* types among ST131 *Escherichia coli* isolates.

**Isolates**	**Phylogenetic group**	***FimH* type**	**Serotype**	**Virotype**	**ESBL genes**	**Fluoroquinolone resistant^***a***^**
5332	B2	*fimH22*	O25:H4	D	*bla_*CMY*−2_*	Pos
7018	B2	*fimH30*	O25:H4	A	*bla_*OXA*−1_*	Pos
7104	B2	*fimH30*	O25:H4	C2	*bla_*KPC*−2_*	Pos
9260	B2	*fimH30*	O25:H4	C	*bla_*CTX*−**M**−15_*	Pos
3218	B2	*fimH30*	O25:H4	C2	*bla_*KPC*−2_*	Pos
9581A	B2	*fimH30*	O25:H4	C	*bla_*CTX*−**M**−15_*	Pos
x5770d	B2	*fimH30*	O25:H4	C	*bla_*CTX*−**M**−15_*	Pos
x6638	B2	*fimH30*	O25:H4	A	*bla_*CTX*−**M**−15_*	Pos
1294D	B2	*fimH30*	O25:H4	B	*bla_*KPC*−2_*	Pos
2102	B2	*fimH30*	O25:H4	A	*bla_*KPC*−2_*	Pos
1710D	B2	*fimH30*	O25:H4	C	*bla_*CTX*−**M**−15_*	Pos
9533D	B2	*fimH30*	O25:H4	C	*bla_*CTX*−**M**−15_*	Pos
3528	B2	*fimH30*	O25:H4	C2	*bla_*CTX*−**M**−15_*	Neg
7078	B2	*fimH30*	O25:H4	C3	*bla_*TEM*−1**B**_*	Pos
9893	B2	*fimH30*	O25:H4	C2	*bla_*KPC*−2_*	Neg
7974	B2	*fimH30*	O25:H4	D	*bla_*CTX*−**M**−2_*	Pos
4233	B2	*fimH30*	O25:H4	NT	*bla_*KPC*−2_*	Pos
5420	B2	*fimH30*	O25:H4	A	*bla_*CTX*−**M**−15_*	Neg
2478	B2	*fimH41*	O16:H5	A	*bla_*TEM*−1**B**_*	Neg
4006	B2	*fimH41*	O16:H5	A	*bla_*TEM*−1**B**_*	Pos
5976	B2	*fimH30*	O25:H4	C3	*bla_*TEM*−1**B**_*	Pos
2206	B2	*fimH30*	O25:H4	A	*bla_*CTX*−**M**−15_*	Pos
8565	B2	*fimH30*	O25:H4	C3	*bla_*TEM*−1**B**_*	Pos
x2724	B2	*fimH30*	O25:H4	C2	*bla_*TEM*−1**B**_*	Pos
6202	B2	*fimH30*	O25:H4	C2	*bla_*TEM*−1**B**_*	Pos
5848	B2	*fimH22*	O25:H4	D	*bla_*CMY*−2_*	Neg

a *Neg. indicates susceptible to fluoroquinolones and Pos. indicates resistant to fluoroquinolones. NT, not typeable*.

**Supplementary data S.6:** Virotypes distribution in ST131 isolates.

**Isolates**	**FimH type**	**Serotype**	***afa*/*draBC***	***afa*operon FM955459**	***iroN***	***sat***	***ibeA***	***papG II***	***papG III***	***cnf1***	***hlyA***	***cdtB***	***neuC-K1***	***kpsM II-K2***	***kpsM II-K5***	**Virotype**
5332	*fimH22*	O25:H4	neg	neg	pos	neg	pos	neg	neg	neg	neg	neg	pos	pos	neg	D
7018	*fimH30*	O25:H4	pos	pos	neg	pos	neg	neg	neg	neg	neg	neg	neg	pos	neg	A
7104	*fimH30*	O25:H4	neg	neg	neg	pos	neg	neg	neg	neg	neg	neg	neg	neg	pos	C2
9260	*fimH30*	O25:H4	neg	neg	neg	pos	neg	pos	neg	pos	neg	neg	neg	pos	neg	C
3218	*fimH30*	O25:H4	neg	neg	neg	pos	neg	neg	neg	neg	neg	neg	neg	neg	pos	C2
9581A	*fimH30*	O25:H4	neg	neg	neg	pos	neg	pos	neg	pos	neg	neg	neg	pos	neg	C
x5770d	*fimH30*	O25:H4	neg	neg	neg	pos	neg	pos	neg	pos	neg	neg	neg	pos	neg	C
x6638	*fimH30*	O25:H4	pos	pos	neg	pos	neg	neg	neg	neg	neg	neg	neg	pos	neg	A
1294D	*fimH30*	O25:H4	neg	neg	pos	pos	neg	neg	neg	neg	neg	neg	neg	neg	pos	B
2102	*fimH30*	O25:H4	pos	pos	neg	pos	neg	neg	neg	neg	neg	neg	neg	pos	neg	A
1710D	*fimH30*	O25:H4	neg	neg	neg	pos	neg	pos	neg	pos	neg	neg	neg	neg	pos	C
9533D	*fimH30*	O25:H4	neg	neg	neg	pos	neg	pos	neg	pos	neg	neg	neg	pos	neg	C
3528	*fimH30*	O25:H4	neg	neg	neg	pos	neg	neg	neg	neg	neg	neg	neg	neg	pos	C2
7078	*fimH30*	O25:H4	neg	neg	neg	pos	neg	neg	neg	neg	neg	neg	neg	neg	neg	C3
9893	*fimH30*	O25:H4	neg	neg	neg	pos	neg	neg	neg	neg	neg	neg	neg	neg	pos	C2
7974	*fimH30*	O25:H4	neg	neg	neg	neg	pos	neg	neg	neg	neg	neg	neg	pos	neg	D
4233	*fimH30*	O25:H4	neg	neg	neg	neg	neg	neg	neg	neg	neg	neg	neg	neg	neg	NT
5420	*fimH30*	O25:H4	pos	pos	neg	pos	neg	neg	neg	neg	neg	neg	neg	pos	neg	A
2478	*fimH41*	O16:H5	pos	pos	neg	neg	neg	neg	neg	neg	neg	neg	neg	pos	neg	A
4006	*fimH41*	O16:H5	pos	pos	neg	neg	neg	neg	neg	neg	neg	neg	neg	pos	neg	A
5976	*fimH30*	O25:H4	neg	neg	neg	pos	neg	neg	neg	neg	neg	neg	neg	neg	neg	C3
2206	*fimH30*	O25:H4	pos	pos	neg	pos	neg	neg	neg	neg	neg	neg	neg	pos	neg	A
8565	*fimH30*	O25:H4	neg	neg	neg	pos	neg	neg	neg	neg	neg	neg	neg	neg	neg	C3
x2724	*fimH30*	O25:H4	neg	neg	neg	pos	neg	neg	neg	neg	neg	neg	neg	neg	pos	C2
6202	*fimH30*	O25:H4	neg	neg	neg	pos	neg	neg	neg	neg	neg	neg	neg	neg	pos	C2
5848	*fimH22*	O25:H4	neg	neg	pos	neg	pos	neg	neg	neg	neg	neg	pos	pos	neg	D

*pos (positive) and neg (negative); Some isolates were'nt possible to subtyping according with Dahbi et al., 2014 were just typing according Blanco et al., 2014, and other were not possible to type being classified as NT, not typeable*.

In the original article, there was an error in the presented percentage of the virotype D among ST131 isolates.

A correction has been made to *Results, Escherichia coli ST131, Paragraph 1*

UPEC strains produce different adhesins and fimbriae, including type 1 fimbriae. The FimH protein is the adhesive subunit of type 1 fimbriae that is used for epidemiological typing of UPEC. In this study, three *fimH* types were identified among the ST131 isolates, two O25:H4/ST131 isolates belonged to *fimH2*2, two O16:H5/ST131 isolates to *fimH*41 while the majority of O25:H4/ST131 isolates (*n* = 22) belonged to *fimH*30 (Table 2). The virulence genes (*afa/draBC, iroN, sat, ibeA, papGII, papGIII, cnf-1, hlyA, cdtB, neuC-K1, kpsMIIK2, kpsmII-K5*) were used to determine the virotype of ST131 isolates based on the virulence profile. O25:H4/ST131 isolates belonged to different virotypes, i.e., 7 (26.92%) to virotype A, 1 (3.84%) to virotype B, 14 (53.84%) to virotype C, and 3 (11.53%) to virotype D. Isolates belonging to virotype C could be divided into subtypes C2 (*n* = 6) or C3 (*n* = 3), whereas five isolates could not be further subtyped. The only two isolates with serotype O16:H5/ST131 were classified as virotype A (see Data Sheet S6). Almost all O25:H4/ST131 isolates were resistant to fluoroquinolones, whereas the O16:H5/ST131 isolates were susceptible to this antibiotic. The *bla*_CTX−M_ gene was most prevalent in O25:H4/ST131 *fimH*30 fluoroquinolones resistant (O25:H4/ST131-H30-R) isolates belonging to virotype C. Within ST131, *bla*_*CTX*−*M*15_ was confined to the H30-R sub-clone known as O25:H4/ST131-H30-Rx, represented by 9 (34.61%) isolates (Table 2).

The authors apologize for these errors and state that this does not change the scientific conclusions of the article in any way. The original article has been updated.

